# Electrical Characteristics of Diamond MOSFET with 2DHG on a Heteroepitaxial Diamond Substrate

**DOI:** 10.3390/ma15072557

**Published:** 2022-03-31

**Authors:** Genqiang Chen, Wei Wang, Fang Lin, Minghui Zhang, Qiang Wei, Cui Yu, Hongxing Wang

**Affiliations:** 1 Key Laboratory for Physical Electronics and Devices, Ministry of Education, Xi’an Jiaotong University, Xi’an 710049, China; genqiangchen@stu.xjtu.edu.cn (G.C.); wei_wang2014@xjtu.edu.cn (W.W.); leaf-lin@mail.xjtu.edu.cn (F.L.); zhangminghuicc@mail.xjtu.edu.cn (M.Z.); wbgwei@xjtu.edu.cn (Q.W.); 2 Institute of Wide Band Gap Semiconductors, School of Electronics and Information Engineering, Xi’an Jiaotong University, Xi’an 710049, China; 3 National Key Laboratory of Application Specific Integrated Circuit, Hebei Semiconductor Research Institute, Shijiazhuang 050051, China

**Keywords:** heteroepitaxial diamond, MOSFET, annealing

## Abstract

In this work, hydrogen-terminated diamond (H-diamond) metal-oxide-semiconductor field-effect-transistors (MOSFETs) on a heteroepitaxial diamond substrate with an Al_2_O_3_ dielectric and a passivation layer were characterized. The full-width at half maximum value of the diamond (004) X-ray rocking curve was 205.9 arcsec. The maximum output current density and transconductance of the MOSFET were 172 mA/mm and 10.4 mS/mm, respectively. The effect of a low-temperature annealing process on electrical properties was also investigated. After the annealing process in N_2_ atmosphere, the threshold voltage (*V_th_*) and flat-band voltage (*V_FB_*) shifts to negative direction due to loss of negative charges. After annealing at 423 K for 3 min, the maximum value of hole field effective mobility (*μ_eff_*) increases by 27% at *V_th_* − *V_GS_* = 2 V. The results, which are not inferior to those based on homoepitaxial diamond, promote the application of heteroepitaxial diamond in the field of electronic devices.

## 1. Introduction

Diamond semiconductors have been studied for decades due to their excellent properties, such as wide band gap energy (5.5 eV), high breakdown electrical field (>10 MV/cm), extremely high thermal conductivity (22 W/cm K), and high carrier mobility (3800 for holes and 4500 cm^2^/Vs for electrons) [1,2,3]. Diamond is preferred for application in high-frequency, high-power electronic devices. Nevertheless, the traditional electronic device fabrication process for diamond is restricted because of the immature n type doping technique. Fortunately, when the hydrogen-terminated diamond (H-diamond) is exposed to air, some adsorbates forming at the H-diamond surface act as electron acceptors. Electrons at the H-diamond surface transfer to these adsorbates so that a two-dimension hole gas (2DHG) is induced [4]. Thanks to diamonds, field effect transistors (FET) have developed greatly [5,6,7]. Up to now, diamond FETs have exhibited excellent performance. K. Ueda et al. achieved a maximum oscillation frequency (fmax) of 120 GHz on high-quality homoepitaxial polycrystalline diamond [8]; Kawarada et al. demonstrated a high-performance H-diamond metal oxide semiconductor (MOSFET) with a maximum output power density of 3.8 W/mm@1GHz [9].

However, to date, the vast majority of diamond electronic devices have been fabricated on HTHP (high temperature and high pressure) and homoepitaxial CVD (chemical vapor deposition) substrates, whose size are greatly limited. This is averse to low cost and commercialization. Thus, in order to resolve this issue, another effective technology should be developed to obtain large and high-quality single crystalline diamond substrates which can reduce costs and enable mass production. It is gratifying that diamond heteroepitaxy on Ir has been extensively developed. Not only the 4-inch size of heteroepitaxial diamond but also the low dislocation density of 9 × 10^6^ cm^−2^ has been achieved [10,11]. Regarding the thick film growth and device fabrication, a-plane sapphire is a promising material to realize large free-standing (001) orientation diamond substrates [12]. Additionally, on this basis, Makoto et al. fabricated Al_2_O_3_/NO_2_/H-diamond MOSFET with an extremely high breakdown voltage on heteroepitaxial single crystal diamond (HSCD) [13].

In this work, we fabricated ALD-Al_2_O_3_/H-diamond MOSFET on a free-standing HSCD. The size of the HSCD is 26 × 26 × 1 mm^3^, and the full-width at half maximum value of the (004) X-ray rocking curve is 209.5 arcsec. The output current density *I_DS_*, maximum transconductance *g_m(max)_* and carrier density of MOSFET with the same size is much better than that we reported previously [14,15], and the effective mobility ($\mu_{\mathrm{eff}}$) and interface states density ($D_{it}$) are discussed pre- and post- annealing process at low temperature in N_2_ atmosphere.

## 2. Materials and Methods

**Figure 1:**
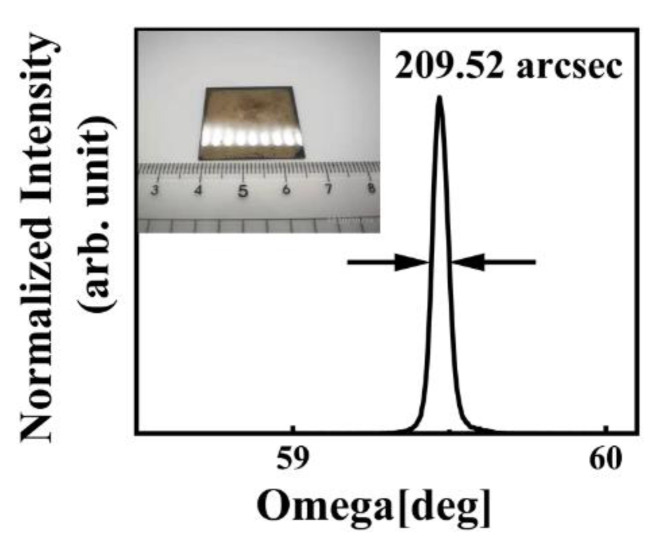
X-ray rocking curve of HSCD.

In this work, an a-plane (11–20) sapphire with a size of 26 × 26 × 1 mm^3^ was chosen as the substrate. Then, approximately 150 nm Ir was deposited at 900 °C using magnetron sputtering technology. Subsequently, bias enhanced nucleation (BEN) was conducted in direct current CVD (Flashforge dreamer©, Jinhua, China) [16]. After the BEN process, diamond epitaxy was carried out in horizontal type MPCVD for 100 h with a growth rate of 10 μm/h. The specific parameters of BEN and epitaxial growth process were reported elsewhere [16]. The insert image is the optical image of a polished heteroepitaxial diamond with a dimension of 26 × 26 × 1 mm^3^. Figure 1 shows the XRD (X-ray diffraction) characteristic of the HSCD. The FWHM of (004) X-ray rocking curve was measured as 209.52 arcsec, which is a relative high value with a size over 1 inch among the heteroepitaxial diamond [10,11,12,17] After cleaning the substrate with mixed acid (HNO_3_:H_2_SO_4_) at 250 °C for 1 h and deionized water in turn, 100 nm homoepitaxial layer was grown on the HSCD with a MPCVD (microwave plasma chemical vapor deposition) system. The growth temperature, pressure, and time were 930~970 °C, 30 Torr, and 60 min. The H_2_ and CH_4_ flow rates were 300 and 0.6 sccm, respectively. The hydrogen plasma was maintained for 20 min to form H-diamond; after stopping CH_4_ flow, 200 nm Au was deposited on the H-diamond surface as source and drain electrodes by the electron beam evaporation technique. Next, ultraviolet ozone (UV/O_3_) was used to convert hydrogen termination into oxygen termination (OT) except for the channel. After that, a 30 nm Al_2_O_3_ passivation layer was deposited on the diamond in two steps by the atomic layer deposition (ALD) technique. Trimethylaluminum (TMA) and H_2_O were adopted as the source and oxidant, respectively. First, a 5 nm Al_2_O_3_ layer was deposited at 90 °C to protect the C-H against oxidation. Second, a 25 nm Al_2_O_3_ layer was deposited at 250 °C. The Al_2_O_3_ on the electrodes was removed by H_3_PO_4_ solution to expose the electrodes for subsequent electrical characterization. Finally, 30/150 nm Ti/Au was deposited on the Al_2_O_3_ layer as the gate electrode. The device characteristics were measured at room temperature (RT).

## 3. Results and Discussion

**Figure 2:**
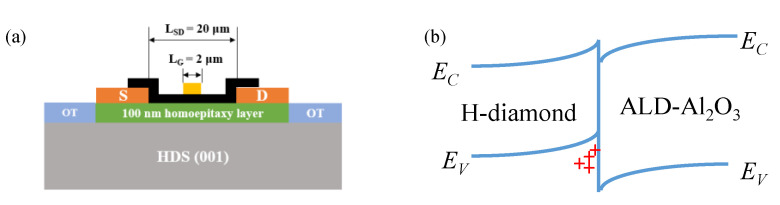
(**a**) Schematic diagram of the MOSFET; (**b**) energy band diagram of H-diamond/Al_2_O_3_ without gate bias.

Figure 2a shows the schematic diagram of the MOSFET. The gate width (*W_G_*), gate length (*L_G_*) and distance source/drain are 100, 2, and 20 μm, respectively. *L_SG_* and *L_GD_* are 9 μm. Figure 2b illustrates the energy band diagram of H-diamond/Al_2_O_3_. The 2DHG under the diamond surface is accumulated due to negatively charged adsorbates, which lead to the energy band’s upward bending at the diamond surface.

**Figure 3:**
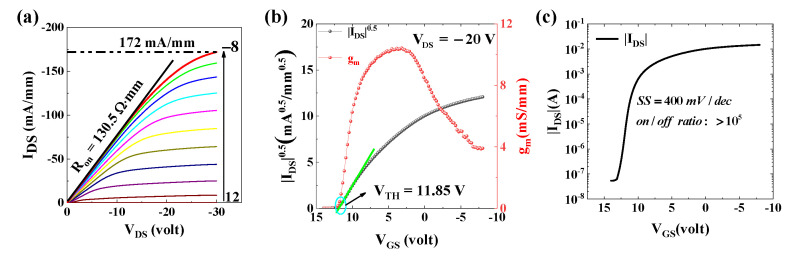
(**a**) Output characteristics of MOSFET; (**b**) transfer curve (**c**) (−I_DS_)-V_GS_ characteristics on a logarithmic scale.

Figure 3 shows the electrical characteristics. The *I_DS_*-*V_DS_* curve is shown in Figure 3a. The gate-source voltage (*V_GS_*) varies from 12 V to −8 V in steps of −2 V. The maximum current density is 172 mA/mm at *V_GS_* = −8 V; *V_DS_* = −30 V. This value is much higher than that we reported based on the homoepitaxial diamond substrate previously [15,16]. It demonstrates the application potential of heteroepitaxy diamond to electronic devices. The on-resistance (*R_ON_*) is evaluated to be 130.5 Ω∙mm. As displayed in Figure 3b, the threshold voltage (*V_th_*) is deduced to be 11.85 V, demonstrating a normally on performance which is obtained according to the relationship of $\sqrt{\left|I_{DS}\right|}$ and *V_GS_*. The maximum transconductance *g_m (max)_* is 10.4 mS/mm. Figure 3c exhibits the relationship of log(|*I_DS_*|) and *V_GS_*. The subthreshold slop and on/off ratio can be observed to be 400 mV/dec and 10^5^.

**Figure 4:**
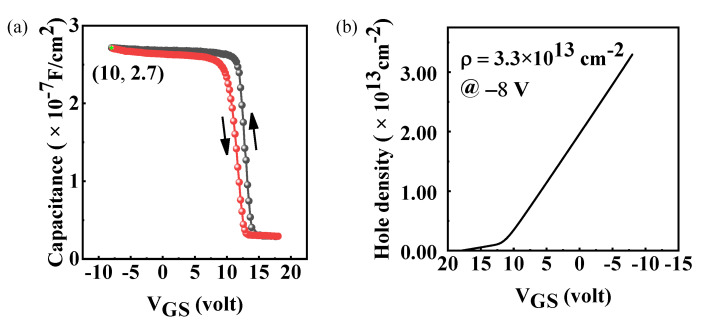
(**a**) C-V curve of Al_2_O_3_/H-terminated MOSFET; (**b**) hole density ρ-*V_GS_* characteristic.

The capacitance voltage (*CV*) characteristics were measured at a frequency of 1 MHz, as displayed in Figure 4a. *V_GS_* swept from 18 to −10 V and −10 to 18 V for the black and red lines, respectively. *C-V* curves shift to the right relative to 0 V, which indicates that negative charges exist in the oxide layer. *C_ox_* for the ALD-Al_2_O_3_ /H-diamond MOS is 2.7 × 10^−7^ F/cm^2^. The flat band capacitance (*C_FB_*) can be calculated from the following equation [18]:

(1) $$C_{FB}=\frac{1}{\left(\frac{1}{C_{ox}}+\frac{L_{D}}{\varepsilon_{0}\varepsilon_{dia}}\right)}$$

where *L_D_* is the Debye length of H-diamond which can be determined to be 2 nm based on carrier concentration of 2 × 10^18^ cm^−3^ for-hydrogen terminated diamond at RT [19], *ε_dia_* is relative permittivity of diamond (5.7). Thus, the *C_FB_* can be calculated to be 2.5 × 10^−7^ F⁄cm^2^. The flat band voltage (*V_FB_*) is determined to be 9.1 and 11.7 V in the reverse (red line) and forward (black line) direction, respectively. The relationship of the fixed negative charge density (*N_fc_*) and flat band voltage can be described as the following equation:

(2) $$N_{fc}=\frac{C_{ox}(V_{FB}+\Delta W/e)}{e}$$

where ∆*W* is the work function difference between H-terminated diamond (4.9 eV) [20] and the Ti (4.3 eV) gate electrode, e is the elementary charge of 1.6 × 10^−19^ C; therefore, the *N_fc_* in Al_2_O_3_ layer can be determined to be 1.66 × 10^13^/cm^2^. The trapped charge density can be calculated to be 4.03 × 10^12^/cm^2^ according to the hysteresis loop (∆*V_FB_* = 2.6 V). Figure 4b shows the relationship between the hole density (ρ) and *V_GS_*. The carrier density can be evaluated by ρ = (∫*C*dV)/e, and the result is 3.3 × 10^13^/cm^2^ obtained at *V_GS_* = −8 V. A linear tendency reveals a uniform carrier distribution of H-diamond.

The field effective mobility *μ_eff_* of the MOSFET can be calculated from the following equation:

(3) $$I_{DS}=\frac{W_{G}\mu_{eff}C_{ox}{\left(V_{GS}-V_{th}\right)}^{2}}{2L_{G}}$$

At RT, the *μ_eff_* is determined to be 36.5 cm^2^/Vs at *V_GS_* = 10 V. For the off-state region, the interface state density *D_it_* can be evaluated from the subthreshold swing *SS*, which is given by [21]

(4) $$SS=\frac{kT(\ln10)}{q}[1+\frac{C_{D}+q^{2}D_{it}}{C_{ox}}]$$

where *k*, T and e are the Boltzman constant, temperature and elementary charge, respectively. *C_ox_* and *C_D_* (*C_D_* ≪ *q*^2^ *D_it_*) are the capacitance of the Al_2_O_3_ layer and depletion layer. The subthreshold swing can be derived from Figure 3c. The minimum *SS* is 400 mV/dec at *V_GS_* = 12 V. Hence, the *D_it_* can be determined to be 1.07 × 10^13^ eV^−1^/cm^2^.

**Figure 5:**
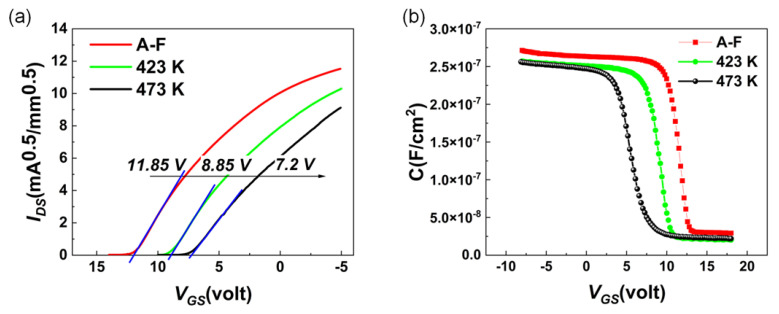
(**a**) transfer curve of MOSFET as-fabricated(A-F) and after annealing process at 423 K and 473 K for 3 min (**b**) *C-V* curve of MOSFET as-fabricated(A-F) and after annealing 150 °C and 200 °C process for 3 min.

**Figure 6:**
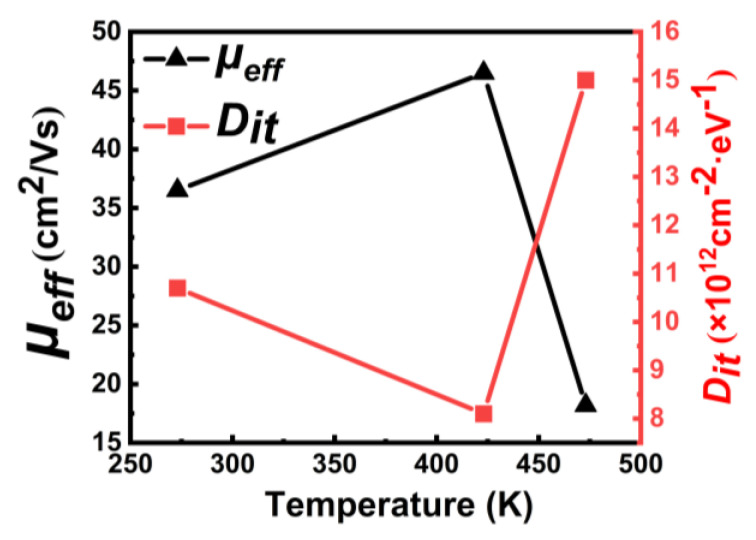
*μ_eff_* at *V_th_* − *V_GS_* = 2 ± 0.2 V and *D_it_* as a function of annealing temperature.

To investigate the effect of low-temperature annealing on electrical properties of the MOSFET, the sample was annealed in N_2_ ambient at 423 K and 473 K for 3 min, sequentially. Threshold voltage (*V_th_*) shifts negatively with increasing annealing temperature, as shown in Figure 5a. In Figure 5b, the *C-V* curve shifts to the negative direction. Additionally, the *V_FB_* for each curve can be extracted to be 9.1 V (red), 6.8 V (green), and 3.3 V (black), respectively. The negatively shifted *V_FB_* indicates that the annealing process decreases the negative charge density at the Al_2_O_3_/H-diamond interface [22]. The loss of negative charges which can induced holes beneath the diamond surface results in negatively shifted *V_th_*, as shown in Figure 5a. Figure 6 presents the *μ_eff_* at *V_th_* − *V_GS_* = 2 ± 0.2 V and *D_it_* as a function of annealing temperature. Obviously, all *μ_eff_* and *D_it_* values show completely inverse trends, which means that *μ_eff_* strongly depends on *D_it_*. When *D_it_* is decreased to 8 × 10^12^ cm^−2^·eV^−1^, the *μ_eff_*, from 36.5 to 46.5 cm^2^/Vs, increases by 27% after the annealing process at 423 K. The possible reason is that when the annealing temperature is 423 K, the quality of ALD-Al_2_O_3_/H-diamond interface was improved. On the contrary, when the annealing temperature increases to 473 K, the *D_it_* increases dramatically and the *μ_eff_* decreases sharply. It implies that 200 °C annealing process for 3 min possibly degrades the interface between Al_2_O_3_ and diamond [22,23]. After the 200 °C annealing process, the increased *D_it_* which acts as charges at interface results in enhancement of coulomb scattering at the ALD-Al_2_O_3_/H-diamond interface.

## 4. Conclusions

In summary, ALD-Al_2_O_3_/H-diamond MOSFETs based on heteroepitaxial diamond substrate were fabricated and characterized. The output current density, carrier density and on-resistance *R_on_* were 172 mA/mm, 3.3 × 10^13^/cm^2^ and 130.5 Ω·mm at *V_GS_* = −8 V, respectively. Both the *V_th_* and *V_FB_* shifted to negative direction, which can be ascribed to loss of fixed negative charges. After annealing at 423 K, *μ_eff_* increased by 27%, accompanying the decreased *D_it_*. Yet, annealing at 473 K for 3 min possibly degrades the Al_2_O_3_/diamond interface. The annealing temperature and period for Al_2_O_3_/H-diamond need to be controlled precisely.

## Data Availability

Data are contained within the article.
